# Monophasic-Quadripulse Theta Burst Magnetic Stimulation for Motor Palsy Functional Evaluation After Intracerebral Hemorrhage

**DOI:** 10.3389/fnint.2022.827518

**Published:** 2022-03-11

**Authors:** Minoru Fujiki, Wataru Matsushita, Yukari Kawasaki, Hirotaka Fudaba

**Affiliations:** Department of Neurosurgery, School of Medicine, Oita University, Oita, Japan

**Keywords:** corticospinal tract, magnetic stimulation, quadripulse theta burst stimulation, multi train stimulation, motor-evoked potentials, quadripulse stimulation, motor palsy

## Abstract

Transcranial magnetic stimulation (TMS) is commonly employed for diagnostic and therapeutic purposes to enhance recovery following brain injury, such as stroke or intracerebral hemorrhage (ICH). Single-pulse TMS, most commonly used for diagnostic purposes and with motor evoked potential (MEP) recordings, is not suitable for clinical use in patients with severe motor paresis. To overcome this problem, we developed a quadripulse theta burst transcranial magnetic stimulation (QTS) device that combines the output from 16 stimulators to deliver a train of 16 monophasic magnetic pulses through a single coil. High-frequency theta rhythm magnetic bursts (bursts of four monophasic pulses, at 500 Hz, i.e., with a 2-ms interpulse interval, repeated at 5 Hz) were generated via a set of 16 separate magnetic stimulators connected to a specially designed combination module. No adverse effects or electroencephalogram (EEGs) abnormalities were identified during or after the recordings. MEP amplification in the QTS during four-burst theta rhythm stimulations produced four independent MEPs 20 ms after each burst onset maximizing the final third or fourth burst, which exhibited significantly greater amplitude than those resulting from a single burst or pulse. Motor functional palsy grades after ICH and QTS-MEP parameters and resting motor threshold (RMT) and amplitudes were significantly correlated (r = −0.83/−0.81 and 0.89/0.87; R^2^ = 0.69/0.66 and 0.79/0.76, *p* < 0.001; anterior/posterior-stimulus polarity, respectively). In conclusion, QTS-MEPs enabled a linear functional evaluation in patients with various degrees of motor paresis. However, the benefits, safety, and limitations of this device should be further explored in future studies.

## Introduction

Transcranial magnetic stimulation (TMS) is commonly employed as an exploratory or diagnostic tool in neuroscience research and for various neurological disorders (Rothwell, 1997; Müller-Dahlhaus and Vlachos, 2013). However, a serious problem with the single-pulse method is the difficulty of inducing reliable and reproducible supramaximal motor evoked potentials (MEPs), especially in awake patients with motor palsy (Deletis and Fernández-Conejero, 2016). In fact, MEP induction rates following TMS are substantially low in patients with severe motor paresis (Rogić et al., 2014). This is because amplification of cortico-muscular MEPs depends on the summation of corticospinal D and I wave descending volleys after a single TMS pulse (Amassian et al., 1990; Di Lazzaro et al., 2003). Furthermore, some clinical situations independent of motor weakness can alter the amplitude of MEPs (Kobayashi et al., 2014; Tamkus et al., 2014). Therefore, a method that enables successful and stable MEP elicitation after TMS in awake patients would be beneficial. To the best of our knowledge, only a specialized coil design for TMS producing an efficient stimulus current in the brain has thus far been developed (Sekino et al., 2015). Alternatively, techniques to amplify MEPs using repetitive multi-train transcranial electrical stimulation (mt-TES) during surgery under general anesthesia in neurologically normal individuals have been described (Szelényi et al., 2007; Deletis and Sala, 2008; Tsutsui et al., 2015; Deletis and Fernández-Conejero, 2016), as have been the optimal repetition conditions of mt-TES in patients with impaired motor function during surgery (Ushio et al., 2018). Theta burst stimulation (TBS) of the human motor cortex (three to five pulses at 100 Hz repeated at 5 Hz), which was originally reported in experimental hippocampal studies for long-term potentiation/depression induction (Hess and Donoghue, 1996), has been successfully translated to awake humans. Patterned repetitive transcranial magnetic stimulation (rTMS) protocols for long-term potentiation/depression-like plasticity induction, which are either intermittent-facilitatory or continuous-inhibitory TBS paradigms for MEPs with rTMS, induce long-term MEP alterations (Huang et al., 2005, 2007). Recently, it has been demonstrated that anterior-posterior (AP) directed continuous quadripulse theta burst magnetic stimulation (QTS) at 666 Hz, with a burst repetition rate of 5 Hz, using 90% active motor threshold-TMS to the hand area of the primary motor cortex, induced an AP-MEP amplitude increase lasting up to 60 min (Jung et al., 2016). Due to the controversial methodological standardization of MEP amplification on the motor cortex either intraoperatively or in awake settings in both humans and animals (Deletis and Sala, 2008; Deletis and Fernández-Conejero, 2016; Sykes et al., 2016; Ushio et al., 2018; Fujiki et al., 2021), a comparison between a facilitatory TBS protocol and standard MEP induction in both awake healthy controls and clinical conditions is needed. Therefore, we developed a monophasic magnetic QTS-induced MEP device system that includes a set of 16 separate magnetic stimulators (Magstim 2002; The Magstim Co. Ltd., Spring Gardens, Whitland, United Kingdom) connected with a specially designed combination module that combines outputs from the 16 stimulators to allow a train of 16 monophasic magnetic pulses to be delivered through a single coil in awake patients with motor deficits. Hence, the total number of pulses and stimulation duration differed among QTS (four bursts, each consisting of four high-frequency monophasic pulses, at 500 Hz, repeated at 5 Hz, for a total of 16 pulses) and two standard MEP protocols (usual number of pulses in each group, four and one). This study aimed to establish a methodology and explore amplification processes during theta rhythm-500 Hz stimulation bursts affecting stable MEP amplification in awake human participants. The configuration of the induced current flow (monophasic polarity, 500 Hz high-frequency bursts at theta rhythm, and motor threshold of stimulus intensity) via a standard figure-8 coil was tested to assess whether it was comparable or superior to the TMS-induced electric fields resulting from a single pulse (SP) or 500-Hz quadripulse single train stimulation of the motor cortex in healthy participants. Determining the protocol reliability and stability for participants with/without motor deficits is important because the present settings offer the potential for application in the near future for speech center brain mapping or exploration of the effects of drugs on the central nervous system (Ziemann et al., 2015) and for the assessment of other pathophysiological conditions. Indeed, the pre-surgical evaluation for surgical indications or continuous recordings for evaluation of motor deficits and acute changes in neurophysiological measures of motor excitability before and after surgical manipulation (Deletis and Sala, 2008) require confirmation by stable and reliable biomarkers. We hypothesized that 500-Hz high-frequency monophasic TBS using TMS will strongly amplify MEPs and demonstrate a direct link between behavioral motor palsy and quantifiable functional parameters. In this regard, we evaluated whether QTS-MEP potentials are quantifiable and beneficial neurophysiological biomarkers of functionally active corticospinal tracts at various degrees of motor paresis after stroke.

The present results provide non-invasive TMS-based novel settings for awake human participants depending on corticospinal pathophysiological conditions.

## Materials and Methods

### Participants and Patients

Ten right-handed healthy men (40–68 years old, mean age ± SD: 58.5 ± 10.8 years) in the control group and 65 hypertensive patients with putaminal intracerebral hemorrhage (ICH) (five women and 60 men; 55–80 years old, mean age ± SD: 68.9 ± 11.8 years) participated in this study. None of the healthy participants had any contraindications to TMS, took any medication on a regular basis, or had a positive history of psychiatric or neurologic diseases (Rossi et al., 2009). All participants provided written informed consent to participate in the study. This study was approved by the Ethics Committee of the School of Medicine, Oita University (protocol number 265). The participants were 65 consecutive patients with impaired motor function, mostly from a compression or destruction of the corticospinal tract by hemorrhage (>5 and <30 ml in volume, with symptom onset <24 h before admission, clear consciousness, no other neurological deficits except for motor dysfunction, and who underwent conservative treatment without surgery between January 2008 and December 2021). The median time from onset to examination was 3.3 (range 1–7) days. The severity of motor function was evaluated according to manual muscle testing (MMT) grades for upper extremities (0, no evidence of contractility; 1, evidence of slight contractility but no joint motion; 2, a complete range of motion with gravity eliminated; 3, a complete range of motion against gravity; 4, a complete range of motion against gravity with some resistance; and 5, a complete range of motion against gravity with full resistance) (Brown and Avers, 2018). The mean upper extremity motor function grade (range, 0–5) was 2.55 ± 1.72 (grade 0, *n* = 10; grade 1, *n* = 12; grade 2, *n* = 11; grade 3, *n* = 12; grade 4, *n* = 10; grade 5, *n* = 10).

### System Configuration and Control Study

**FIGURE 1:**
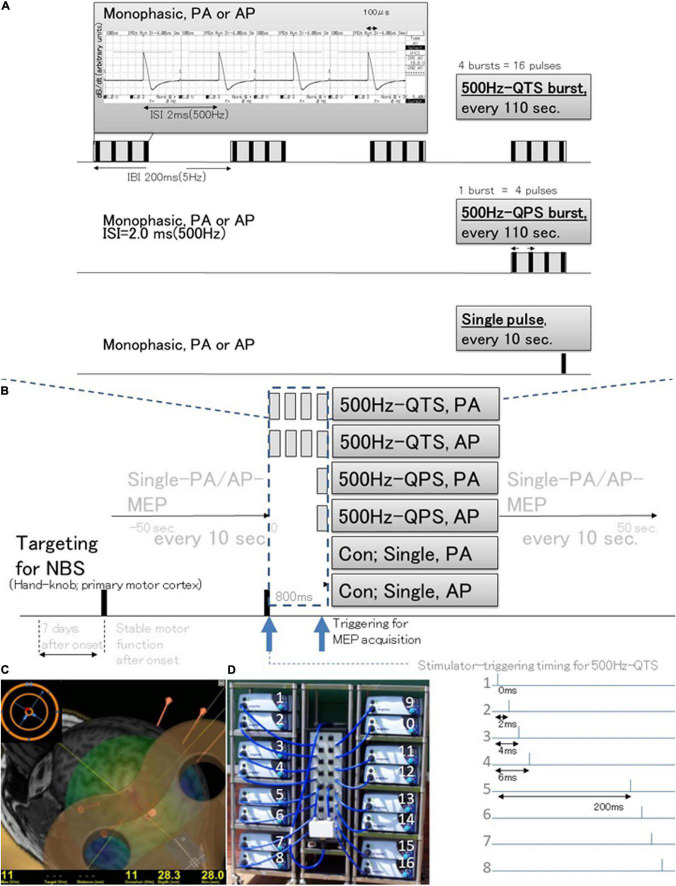
Schematic illustration of the experimental protocol and stimulus configurations. Comparison between standard single-pulse magnetic stimulation induced-MEPs, single 500-Hz burst and four 500-Hz bursts, repeated at 5-Hz stimulation MEPs (different induced current frequencies, current flow configuration in the motor cortex, and polarity; monophasic PA and AP directions in each modality). **(A)** Stimulus conditions: 500-Hz quadripulse theta burst stimulation (QTS), 500-Hz quadripulse single train stimulation (QPS), and monophasic single pulse. PA and AP polarity in each modality were compared. QTS consisted of four bursts, each consisting of four high-frequency monophasic pulses, at 500 Hz (i.e., 2 ms-ISI), repeated at 5 Hz (i.e., 200-ms interburst interval), and delivered continuously for 800 ms, with a total of 16 pulses. QPS consisted of four high-frequency monophasic pulses delivered at 500 Hz [i.e., 2-ms interstimulus interval (ISI)]. MEP acquisitions were performed from the final burst. An analysis window of 800 ms was applied in QTS for the acquisition of MEP development after each burst during stimulation only in healthy controls (dark blue dashed box). **(B)** SP, QPS, or QTS were applied to the hand area of the left motor cortex with a PA- or AP-directed monophasic magnetic QTS-induced MEPs device system that includes a set of 16 separate magnetic stimulators (Magstim, 2002; The Magstim Co. Ltd., Wales, United Kingdom) connected with a specially designed combining module (2020-036704). **(C)** MEPs were recorded under the targeted primary motor cortex for the first dorsal interosseous (FDI) muscle using the navigated brain stimulation system within 7 days after onset. The screenshot depicts a representative control case with an optimized see-through coil and the area mapped to identify the motor optimal location (hotspot) in the target muscle. Each dot on the scalp can be visualized as orange balls and the red arrow shows the current direction of pulses in the brain. The colors show the relative strength of the E-field (red, high E-field strength; blue, low E-field strength). The position feedback indicator (small window on the right for repeated constant stimulation) providing real-time feedback surface location-enabled manual holding-reliable targeting. **(D)** The QTS-induced MEPs device system consisted of a set of 16 separate magnetic stimulators (Magstim, 2002; The Magstim Co. Ltd., Wales, United Kingdom) connected with a specially designed combining module (patent number: 2020-036704). This device combines the outputs from 16 stimulators to deliver a train of 16 monophasic magnetic pulses through a single coil. QTS, QPS, or SP were applied to the hand area of the left motor cortex with a PA- or AP-directed monophasic pulse. QTS, quadripulse theta burst stimulation; QPS, quadripulse stimulation; PA, posterior-anterior; AP, anterior-posterior; ISI, interstimulus interval; IBI, interburst interval; RMT, resting motor threshold; TMS, transcranial magnetic stimulation; RMT, resting motor threshold; MEP, motor evoked potential; NBS, navigated brain stimulation.

Control studies for healthy participants were performed to test six different configurations of the induced current flow [i.e.,monophasic SP, 500 Hz quadripulse single train stimulation (QPS), 500-Hz QTS, and polarity in each modality via a 70 mm figure-8 coil delivering magnetic pulses at 1.2 times the resting motor threshold (RMT) of the MEPs targeting the primary motor cortex for the first dorsal interosseous (FDI) muscle with a navigated brain stimulation system (optical tracking system that enables real-time precise TMS tracking, see Figure 1C for details; Nexstim eXima; Nexstim Ltd., Helsinki, Finland)]. SP, QPS, and QTS were applied to the hand area of the left motor cortex with a posterior-anterior (PA)- or AP-directed monophasic magnetic QTS-induced MEP device system, a set of 16 separate magnetic stimulators (Magstim, 2002; The Magstim Co. Ltd., Wales, United Kingdom) was connected to a specially designed combination module (Figure 1; patent number: 2020-036704). This device combines the outputs of 16 stimulators to deliver a train of 16 monophasic magnetic pulses through a single coil. The basic procedures of single-pulse MEP recordings followed previously described methods (Hamada et al., 2013; Nakamura et al., 2016). Briefly, the RMT was determined by first decreasing the stimulator output to the 1% maximum stimulator output until MEPs disappeared and then increasing the output in 1% maximum stimulator output increments until six 50 μV MEPs (peak-to-peak) were elicited out of every 12 pulses. MEPs were measured using Ag/AgCl cup electrodes on the right FDI muscle. Subsequently, they were pre-amplified and stored (Neuromaster, Nihon-Kohden Co. Ltd., Tokyo, Japan and Brain Vision Recorder, Brain Products, Germany, with 5–3,000 Hz band pass at a sampling rate of 5,000 Hz and 100-ms analysis window). The first 100 ms of electromyography data after stimulation was used for quantification.

**FIGURE 2:**
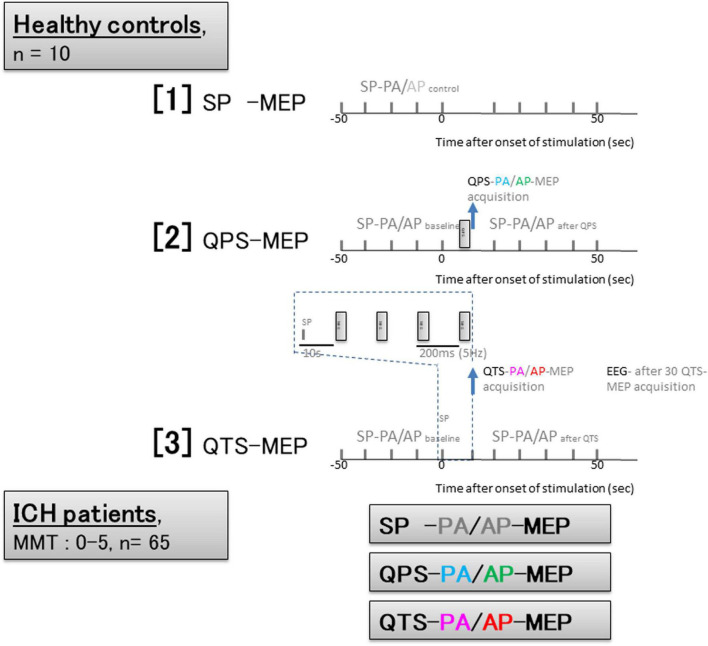
Experimental protocol based on time series configurations for healthy controls and patients with ICH. Healthy controls; [1] Ten SP-PA and AP- motor evoked potential (MEP) were recorded every 10 s. [2] quadripulse theta burst stimulation (QPS)- and QTS-PA and AP-MEPs were recorded every 110 s. [3] QTS-MEP acquisitions were triggered from the beginning of the first burst and continued to the end of the fourth and final burst to verify MEP amplification in QTS during four-burst theta rhythm stimulations (analysis window of 800 ms; dark blue dashed box). SP-PA or AP-MEPs were recorded every 10 s before and after 50 s. QTS-MEPs after the fourth burst were measured to detect RMT and measure amplitude and latency. Electroencephalograms (EEGs) were recorded for ≥30 min post-recording in 10 participants after 30 QTS. Patients with ICH; Trigger for MEP acquisition: QTS-MEP after the fourth burst, QPS-MEP after single burst, and SP-MEPs were measured to detect RMT and measure amplitude and latency in each patient. Sessions without stable recording after 30 trials were interrupted.

The QPS consisted of four high-frequency monophasic pulses delivered at 500 Hz [i.e., 2-ms interstimulus interval (ISI)]. The QTS consisted of four bursts, each consisting of four high-frequency monophasic pulses, with a pulse frequency of 500 Hz (i.e., 2-ms ISI), repeated at 5 Hz (i.e., 200-ms interburst interval), and delivered continuously for 800 ms, for a total of 16 pulses. MEP acquisitions were triggered from the beginning of the first burst and continued to the end of the fourth-final burst to verify MEP amplification in QTS during four-burst theta rhythm stimulations (analysis window of 800-ms; dark blue dashed box, Figures 1A,B,D, 2). Stable and reproducible hand muscle-MEPs were employed instead of forearm-recording, which may reflect MMT grades. After 10 continuous SP-PA and AP-MEP recordings every 10 s, QPS and QTS-PA and AP-MEPs were recorded every 110 s. Since SP-MEPs fluctuated for several seconds immediately after QTS in the preliminary study, SP-PA or AP-MEPs were recorded every 10 s before and after 50 s for stability verification (healthy controls are shown in Figure 2). QTS-MEPs after the fourth burst were measured to detect RMT and measure amplitude and latency. Such stimulation yielded MEPs from the hand FDI muscle when the motor cortex was stimulated at the hand knob of the primary motor cortex (Hannula et al., 2005). For subclinical abnormality detection, electroencephalograms (EEGs) were recorded using Brain-Vision-Recorder [TMS-compatible 32 channel electrodes based on the international 10–20 system (Cz, C1, C3, C5, FCz, FC1, FC3, FC5, CPz, CP1, CP3, and CP5), bandpass-filtered between 0.1 and 500 Hz, and sampled at the rate of 1,450 Hz; Brain-Products, Germany] for ≥30 min post-recording in ten participants. To verify the long-lasting aftereffect of QTS, SP-MEP baseline parameters (RMT, latency, and amplitude) were compared with the baseline after 30 QTS trials (480 pulses, total).

### Magnetic Motor Cortex Stimulation and Motor Evoked Potential Recording for Patients With Intracerebral Hemorrhage

The basic procedures of magnetic stimulation and MEP recordings for patients with ICH were based on the methods for healthy controls described above. Since it is difficult to identify the primary motor cortex in participants with motor weakness or paralysis by MEP alone, stimulus points were determined using the navigated brain stimulation system, and then, the appropriate stimulus intensities for RMT were explored in SP, QPS, and QTS. The figure-8 coil was attached to the skull so that PA- or AP-directed induced currents in the brain were perpendicular to the hand knob of the primary motor cortex (Hannula et al., 2005). To assay descending motor systems, we stimulated the motor cortex of the affected side and measured the MEPs from the contralateral FDI muscle. For motor cortex stimulation, SP, QPS, and QTS PA- or AP-directed monophasic wave pulses were delivered through a single figure-8 coil to achieve temporal summation for selective motor cortex activation. RMTs in patients with severe motor palsy were determined until 100% maximum stimulator output was reached and so that six 50 μV MEPs (peak-to-peak) were elicited out of every 12 trials. Sessions without stable recording after 30 trials were interrupted. QTS-MEP acquisitions were triggered from the end of the fourth-final burst to evaluate corticospinal function in patients with ICH (Figure 2). For testing, trains of stimuli were delivered every 10 s (SP) to 110 s (QPS and QTS) to allow for response recovery (Fujiki et al., 2021).

### Data Analysis

Motor evoked potential data were analyzed offline and assessed visually by a single experienced professional (MF), using Brain-Vision-Analyzer2 (Brain-Products, Munich, Germany), similar to previous reports (Sykes et al., 2016; Fujiki et al., 2021). Latencies and peak-to-peak MEP amplitudes were measured (at 120% RMT-intensity, i.e., six-individual-sweeps in each-minute run). MEPs below 50 μV were regarded as “not recordable” and excluded because a substantial MEP amplitude is required for reliable motor functional evaluation. Amplitudes were normalized to the MEP amplitude measured at the baseline and expressed as a percentage change, allowing for between-subject comparisons.

All data are presented as mean ± SD. For QTS and QPS amplification effects in healthy participants, the statistical significance of group differences was analyzed by ANOVA with time (TIME) as a within-subject factor and group (GROUP) as a between-subject factor (SPSS, Cary, NC, United States). This was followed by a *post hoc* Holm test. To investigate whether the time effect differed among groups, we confirmed the TIME × GROUP interaction. Differences were considered significant at *p* ≤ 0.05. Multiple comparisons of different stimulating configurations were analyzed with RMT and MEP amplitudes using two-way repeated-measures ANOVA [between-subject factor, STIMULUS CONDITION (SP-PA, SP-AP, QPS-PA, QPS-AP, QTS-PA, and QTS-AP); within-subject factor, MMT grades (5–0)]. This was followed by *post hoc* Bonferroni’s corrections for multiple comparisons. The correlation coefficient (r) and coefficient of determination (R^2^) were calculated to assess whether the motor function of the upper extremities after ICH correlated with MEP parameters, RMTs, and amplitudes of each recording.

## Results

### Motor Evoked Potential Amplification During Four 500 Hz High-Frequency Burst Stimulations in Healthy Controls

None of the participants reported any adverse effects during or after the recordings. No EEG abnormalities were found during or ≥30 min post-recording.

**FIGURE 3:**
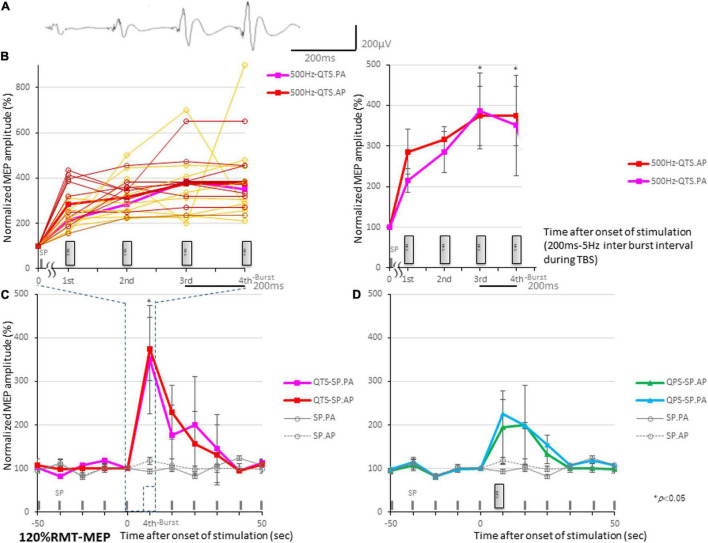
Motor evoked potential (MEP) amplification during four 500-Hz high-frequency burst stimulations. **(A)** MEP amplification in quadripulse theta burst transcranial magnetic stimulation (QTS) during four-burst stimulations at theta rhythm in a healthy participant. Four independent MEPs were generated 20 ms after each burst onset [four pulses at 500 Hz (2-ms ISI), repeated at 5 Hz (200-ms interburst interval)]. MEP amplitudes linearly increased after each burst, finally reaching 350–370% of a single-pulse-MEP. **(B)** MEP amplification during 500-Hz QTS-PA and -AP, respectively. Four gray boxes designate each 500-Hz burst [individual trace overlay (PA: orange) and (AP: brown); left and averaged trace (PA: pink) and (AP: red); right]. Time-series data of MEP amplitudes expressed as percentage change from baseline attributable to TBS. Group data are presented as mean ± standard deviation. Analysis time and a time window of 800 ms were applied in QTS for MEP development acquisition after each burst during stimulation. **(C)** SP-MEP time course recorded at 10-s intervals before and after 500-Hz QTS (dark blue dashed box indicates time windows during QTS, corresponding to part B). **(D)** SP-MEP time course recorded at 10-s intervals before and after single 500-Hz QPS, PA, and AP-direction, respectively. SP-PA and AP-MEP control time courses are exhibited as gray-solid and dashed lines, respectively.

Motor evoked potential amplification in QTS during four-burst theta rhythm stimulations is illustrated in Figure 3A. Four independent MEPs were generated 20 ms after each burst onset [four pulses at 500 Hz (2-ms ISI), repeated at 5 Hz (200-ms interburst interval)]. MEP amplitudes were linearly increased after each burst, finally reaching 350–370% of the baseline-single-pulse MEP. Figure 3B illustrates MEP amplification during 500 Hz-QTS PA and AP, respectively [individual trace overlay (PA: orange) and (AP: brown); left and averaged trace (PA: pink), and (AP: red); right]. Four gray boxes indicate each 500-Hz burst. Figure 3C illustrates the SP-MEP time course recorded at 10-s intervals before and after 500 Hz-QTS (dark blue dashed box indicates 800 ms-time windows during QTS, corresponding to Figure 3B). Figure 3D illustrates the SP-MEP time course recorded at 10-s intervals before and after single 500 Hz-QPS, PA, and AP-direction, respectively. SP-MEP immediately after QTS and QPS was fluctuated with higher amplitudes compared to baseline, and then was gradually decreased, returning to near-control levels by 30 s. SP-PA and AP-MEP control time courses were indicated by gray solid and dashed lines, respectively. An ANOVA for Figure 3B–reveals a significant main effect of MEP after QTS, whereby the effects of stimulation differed among the four groups [main effect of GROUP, *F*_(3,33)_ = 20.61, *p* < 0.001; main effect of TIME, *F*_(9,302)_ = 14.17, *p* < 0.001; and interaction of GROUP × TIME, *F*_(21,302)_ = 2.91, *p* < 0.001].

A *post hoc* analysis indicated significant increases, compared with the baseline and following SP-MEP, in the MEP amplitudes after stimulation in QTS PA and AP (*p* < 0.001). Multiple comparisons between the QTS and QPS were conducted at each time point. Our results indicated the MEP amplitudes in QTS PA and AP were significantly increased at the final time points (at the third burst and fourth burst: *p* < 0.001, respectively; Figures 3B–D).

### Characteristic Resting Motor Threshold and Amplitude Profiles of Quadripulse Theta Burst Transcranial Magnetic Stimulation-Motor Evoked Potentials in Healthy Controls

**FIGURE 4:**
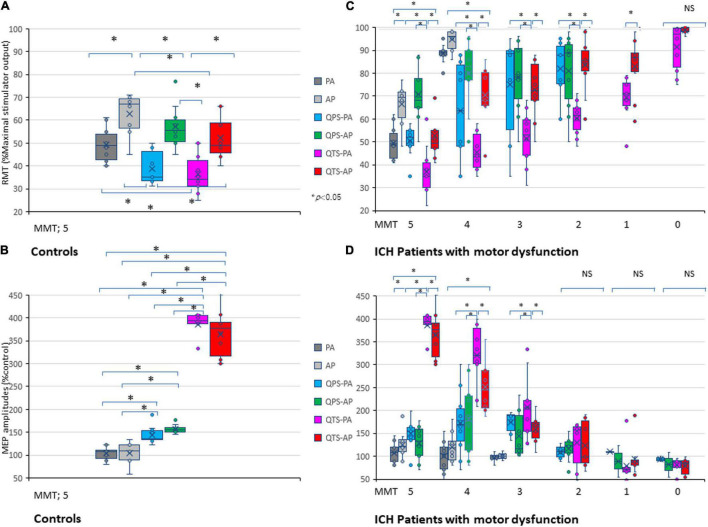
Five hundred-Hertz quadripulse theta burst transcranial magnetic stimulation (QTS), 500 Hz-single burst, and single pulse: comparison of characteristic resting motor threshold (RMT) and amplitude profiles, and the correlation between manual muscle testing (MMT) grades. Quantitative differences in motor evoked potential (MEP) baseline parameters between the six stimulus conditions were statistically significant in RMT **(A)** and amplitude **(B)** in healthy controls (*p* < 0.05). RMTs were significantly higher in the AP direction in all QTS, QPS, and SP configurations (*p* < 0.05). A multiple comparison test revealed significant differences in RMT and MEP amplitudes, and that the QTS induces higher amplitudes with lower stimulus intensities in healthy controls (*p* < 0.05). Significant differences were found in RMT (MMT grades 5–1) and amplitude (MMT grades 5–3) in patients with ICH [Panels **(C,D)**; *p* < 0.001]. There were significant correlations between MMT neurological grades and RMTs, and QTS-PA/AP-MEP amplitudes (*p* < 0.001, respectively), but not those of QPS-AP/PA or single-pulse-PA/AP. Colors in the graph represent each condition before and after TMS [red: 500-Hz QTS-AP, pink: 500-Hz QTS-PA; green: 500-Hz QPS-AP; light blue: 500-Hz QPS-PA; dark gray: single-pulse PA; light gray: single-pulse-AP in healthy controls **(A,B)** and MMT-parameter correlations in patients with ICH **(C,D)**, respectively]. *p < 0.05.

Single pulse-, QPS-, and QTS-induced MEPs in healthy controls were compared for the validation of characteristics (different methodological configurations of the induced current frequency current flow in the motor cortex and polarity; monophasic PA and PA directions in each modality). MEP amplifications during monophasic 500-Hz high-frequency theta rhythm burst repetition were compared with single 500-Hz QPS burst MEP and single-pulse MEP (see Figures 2, 3 for details). One way ANOVA revealed significant differences in MEP parameters between the six stimulus conditions in RMT and amplitude [RMT: *F*_(5,54)_ = 14.38 *p* < 0.001, amplitude: *F*_(5,54)_ = 217.3, respectively, *p* < 0.001; Table 1], while no statistical difference was found in latency [*F*_(5,54)_ = 0.68, *p* = 0.640]. A *post hoc* multiple comparison analysis indicated a significant difference in the RMT and MEP amplitudes between the stimulus conditions (*p* < 0.05; Figures 4A,B). For the verification of QTS long-lasting aftereffect, no significant differences in SP-MEP parameters after 30 QTS trials were observed when compared with baseline (RMT: 50.7 ± 1.92 vs. 50.9 ± 1.95%; latency: 22.75 ± 0.59 vs. 22.01 ± 0.34 ms; amplitude: 284.3 ± 12.03 vs. 307 ± 23.27 μV; after SP-control and QTS, respectively). In addition, QTS had no significant effects on RMT [*t*_(18)_ = − 0.07; *p* > 0.05], latency [*t*_(18)_ = 1.08; *p* > 0.05], or amplitude [*t*_(18)_ = − 0.89; *p* > 0.05].

**TABLE 1:** Quantitative differences in the motor evoked potential (MEP) parameters between six stimulus conditions.

MMT		Single SP-PA	SP-AP	QPS QPS-PA	QPS-AP	QTS QTS-PA	QTS-AP	*F*	*P*
**Healthy controls**									
5 (*n* = 10)	RMT (%)	49.2 ± 7.71	62.7 ± 8.72	38.9 ± 7.23	57.1 ± 9.19	36.5 ± 8.83	52.4 ± 9.24	*F*_(5,54)_ = 14.38	**<0.001**
	Amplitude (μV)	104.1 ± 14.37	104.5 ± 22.58	143.9 ± 19.11	157.1 ± 9.19	385.6 ± 28.89	364.9 ± 51.41	*F*_(5,54)_ = 217.3	**<0.001**
	MEP recordable	10	10	10	10	10	10		
**ICH patients**									
5 (*n* = 10)	RMT (%)	49.4 ± 7.54	66.4 ± 8.84	50.5 ± 6.67	70.7 ± 11.87	36.9 ± 10.91	52.6 ± 9.73	*F*_(5,54)_ = 17.03	**<0.001**
	Amplitude (μV)	107.3 ± 22.71	124.8 ± 29.11	148.9 ± 30.84	128 ± 37.53	385.6 ± 28.85	364.9 ± 51.41	*F*_(5,54)_ = 138.5	**<0.001**
	MEP recordable	10	10	10	10	10	10		
4 (*n* = 10)	RMT (%)	88.6 ± 4.03	94.7 ± 4.29	63.4 ± 22.12	80.1 ± 15.04	45.6 ± 8.07	70.3 ± 12.03	*F*_(5,54)_ = 20.14	**<0.001**
	Amplitude (μV)	99.6 ± 33.78	119.3 ± 32.05	172.9 ± 70.58	180.1 ± 79.09	321 ± 66.56	251.1 ± 54.52	*F*_(5,54)_ = 19.75	**<0.001**
	MEP recordable	10	10	10	10	10	10		
3 (*n* = 12)	RMT (%)	100	100	72.8 ± 22.29	75.8 ± 15.33	49.8 ± 11.94	70.8 ± 12.57	*F*_(3,49)_ = 9.843	**<0.001**
	Amplitude (μV)	97.8 ± 12.48	100.3 ± 10.51	172.8 ± 22.29	145.6 ± 49.75	197.4 ± 75.41	158.6 ± 28.25	*F*_(3,49)_ = 5.063	**<0.001**
	MEP recordable	4 (33.3%)	3 (25%)	12	12	12	12		
2 (*n* = 11)	RMT (%)	>100	>100	81.3 ± 13.37	81.7 ± 16.54	60.4 ± 9.15	83.9 ± 12.09	*F*_(5,38)_ = 5.073	**0.0012**
	Amplitude (μV)	Not recordable	Not recordable	108.1 ± 13.12	116.2 ± 25.44	133.1 ± 51.14	129.3 ± 50.07	*F*_(5,38)_ = 0.543	0.742
	MEP recordable	0	0	11	11	11	11		
1 (*n* = 12)	RMT (%)	>100	>100	100	100	69.5 ± 9.15	79.5 ± 12.09	*F*_(5,22)_ = 4.518	**0.005**
	Amplitude (μV)	Not recordable	Not recordable	109.5 ± 2.12	89.4 ± 48.08	77.5 ± 35.68	87.9 ± 35.68	*F*_(5,22)_ = 0.326	0.992
	MEP recordable	0	0	2 (16.7%)	2 (16.7%)	12	12		
0 (*n* = 10)	RMT (%)	>100	>100	100	100	91.4 ± 10.25	98.7 ± 13.37	*F*_(5,11)_ = 1.762	0.201
	Amplitude (μV)	Not recordable	Not recordable	94.8 ± 8.48	81.5 ± 37.47	80.71 ± 15.38	78.3 ± 19.05	*F*_(5,11)_ = 0.176	0.966
	MEP recordable	0	0	2 (20%)	2 (20%)	7 (70%)	6 (60%)		

*MEP, motor evoked potential; RMT, resting motor threshold; QTS, quadripulse theta burst stimulation; QPS, quadripulse stimulation; SP, single pulse; PA, posterior-anterior; AP, anterior-posterior. Bold indicates P-value < 0.05.*

### Correlation Between Manual Muscle Testing Grade and Quadripulse Theta Burst Transcranial Magnetic Stimulation-Motor Evoked Potential Parameters in Patients With Motor Dysfunction After Intracerebral Hemorrhage

Age (68.9 ± 11.8) and hematoma volume (5–28 ml, with a mean of 10.2 ± 5.58) did not correlate with MMT grades in patients with ICH.

Among the six stimulus conditions in six MMT groups (SP-PA/AP, QPS-PA/AP, and QTS-PA/AP in each MMT 5–0 groups), there were significant differences in RMT for MMT grades 5–1 and amplitude for MMT grades 5–3 in patients with ICH (*p* < 0.001; Table 1). Multiple comparisons among the six conditions and seven groups (i.e., healthy controls) were conducted for each MMT grade. One way ANOVA revealed significant differences in MEP parameters between the six stimulus conditions in RMT and amplitude [RMT: *F*_(34_,_287)_ = 23.37 *p* < 0.001, amplitude: *F*_(34_,_287)_ = 53.87, respectively, *p* < 0.001]. Two-way repeated-measures ANOVA revealed a significant CONDITION (SP-PA/AP, QPS-PA/AP, and QTS-PA/AP) × MMT interaction [RMT: *F*_(23,287)_ = 3.29, *p* < 0.001, amplitude: *F*_(23,287)_ = 17.11, *p* < 0.001, respectively]. A *post hoc* multiple comparison analysis indicated significant differences in the RMT (between QTS-PA and QTS-AP; QTS-AP and QPS-PA, SP-PA, respectively) and MEP amplitudes (between QTS-AP and QPS-PA/AP, SP-PA/AP, respectively, *p* < 0.001; Figures 4C,D).

**FIGURE 5:**
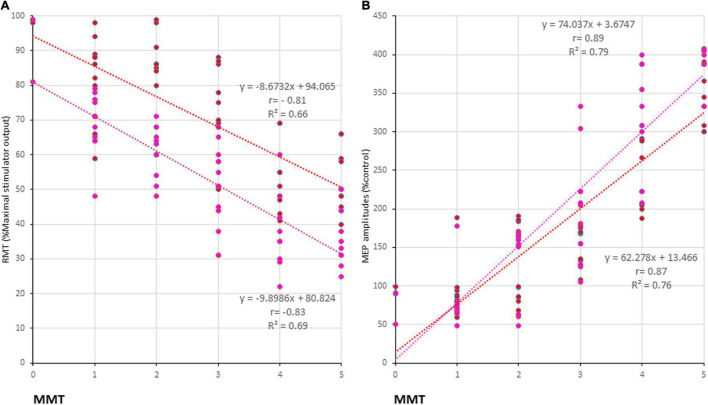
Correlation between manual muscle testing (MMT) grades and physiological parameters of 500-Hz quadripulse theta burst transcranial magnetic stimulation (QTS) motor evoked potential (MEP). There were significant correlations between MMT neurological grades and RMTs **(A)**, and amplitudes **(B)** of QTS-PA/AP-MEP (*p* < 0.001, respectively). Colors in the graph represent each condition before and after Theta burst stimulation [TBS; red: 500 Hz QTS-AP, pink: 500 Hz QTS-PA in patients with intracerebral hemorrhage (ICH), respectively].

The motor function of the upper extremities (as indicated by the MMT grades), RMTs, and amplitudes of QTS-MEPs were significantly correlated [RMT: *r* = −0.83, R^2^ = 0.69, *p* < 0.001/*r* = − 0.81, R^2^ = 0.66, *p* < 0.001; amplitudes: r = 0.89, R^2^ = 0.79, *p* < 0.001/r = 0.87, R^2^ = 0.76, *p* < 0.001, QTS-PA/AP polarity, respectively; Figures 4C,D; pink (PA) and red (AP) line in Figures 5A,B, respectively], but that was not the case for QPS-AP/PA or single-pulse-PA/AP.

## Discussion

We validated various configurations of QTS-MEP amplification by two different MEP-eliciting magnetic stimulations of the motor cortex in both healthy controls and patients with ICH. For our study, we employed a similar stimulation pattern for MEP facilitation both in patterned TMS for LTP induction and mt-TES for intraoperative MEP, as in previous human studies [patterned TMS for LTP induction: QTS; Jung et al. (2016), QPS5; Hamada et al. (2013) mt-TES for intraoperative MEP; Szelényi et al. (2007); Deletis and Sala (2008), Tsutsui et al. (2015), and Deletis and Fernández-Conejero (2016), respectively]. We delivered our pulses at a high-frequency 500-Hz burst, repeated at 5 Hz (four pulses with a 2-ms interpulse interval; QTS) every 110 s, and followed by every 10 s (monophasic SP; SP), with various pulse numbers and different frequencies and durations, in each condition.

Compared with standard PA- or AP-directed SP-MEP, we found that repetitive high-frequency magnetic stimulation at 500 Hz, repeated at 5 Hz, strongly amplified MEPs, recovering to baseline values within 50 s.

### Five Hundred-Hertz Quadripulse Theta Burst Transcranial Magnetic Stimulation of the Motor Cortex Facilitates Motor Evoked Potential

It is notable that both PA and AP-QTS induce 350–370% of single-pulse MEP amplitudes with significantly low stimulus intensities. Four monophasic pulses at 500 Hz repeated four times at 5-Hz configurations affected MEP amplification when compared with SP. On the other hand, considering the difference between QTS-MEP and patterned rTMS protocols for LTP, such as QPS or continuous theta burst QTS (while stimulus intensity current density is higher but shorter duration, it has a lower intensity but longer duration with patterned rTMS protocols), QTS stimulus parameters for MEP amplification may not always induce the same neuroplasticity as that in patterned rTMS (Hoogendam et al., 2010). In fact, QTS-amplified MEP amplitudes were recovered within 10–50 s. Furthermore, different stimulus configurations for QTS-MEP amplification have never been applied for stable MEP induction in the motor cortex of awake humans and animals, except for intraoperative use with transcranial electrical stimulation under general anesthesia (Szelényi et al., 2007; Deletis and Sala, 2008; Tsutsui et al., 2015; Ushio et al., 2018). Comparative experimental settings with other patterns of stimulus configurations will help to clarify the underlying mechanisms and verify the compatibility between human and animal results. However, the fact that QTS appeared to induce stronger facilitation than QPS in healthy participants is interesting. In this regard, QTS-MEP amplitude evoked 20 ms after the fourth burst with lower stimulus intensity than other stimulus configurations suggests the higher recruitment gain by QTS. Short-lasting SP-MEP fluctuations by 30 s after QTS or single QPS suggest that the short-term plasticity processes as a possible interpretation, but this was not verified in the present study. It is possible that the burst repetition rate at 5 Hz provides stronger developing phases in QTS (QTS; four monophasic pulses at 500 Hz repeated four times at 5 Hz vs. single burst) (Jung et al., 2016). Indeed, it is possible that a 500 Hz-ISI (i.e., 2-ms interpulse interval) facilitates MEPs similarly by the temporal summation of corticospinal D and I waves (Deletis and Sala, 2008; Deletis and Fernández-Conejero, 2016). In contrast, the fundamental mechanism of the 5-Hz interburst interval in TBS-MEP facilitation remains uncertain (Jung et al., 2016). Future research in both healthy and symptomatic participants is warranted.

### Resting Motor Threshold and Amplitude of 500-Hz Quadripulse Theta Burst Transcranial Magnetic Stimulation-Motor Evoked Potentials as Surrogate Quantitative Biomarkers for Intracerebral Hemorrhage

The two electrophysiological parameters (RMT and amplitude of the 500-Hz QTS-MEPs in both the PA and AP current directions) found to correlate with neurological MMT grades could be useful as surrogate neurophysiological quantitative biomarkers. Single-pulse TMS of the motor cortex induces 2-ms periodical descending volleys, which results in temporal summation that generates cortico-muscular MEPs under healthy conditions (Amassian et al., 1990; Di Lazzaro et al., 2003). The difficulty of inducing reliable and reproducible SP-TMS-MEPs in patients with motor palsy potentially has been solved by the ability to elicit MEPs after 500-Hz QTS, which is an important contribution of the present study. Furthermore, there were significant correlations between the QTS-MEPs and MMT scores, specifically with a higher correlation for MEPs amplitude than RMTs. Notably, the 500-Hz QTS-MEP alone exhibited linear correlations. To understand the underlying mechanisms, potential pathophysiological background and exploration of corticospinal excitability after stroke using 500-Hz QTS will be helpful. However, since we did not perform simultaneous quantitative morphological measurements, such as diffusion tensor tractography, the present results might not directly reflect the degree of spared active corticospinal tracts within 7 days after ICH. Careful interpretation is required because both MMT grades and MEP parameters are non-linear (Di Lazzaro et al., 2003; Deletis and Sala, 2008; Deletis and Fernández-Conejero, 2016).

### Limitations and Future Work

First, the present study recruited only patients with ICH, which comprise a lower proportion of stroke patients compared to ischemic stroke patients (Virani et al., 2021). To understand the underlying mechanisms and to verify compatibility and/or differences between ischemic and hemorrhagic strokes, further studies with the same experimental strategy are warranted. On the other hand, since the patients in the present study were under acute stroke conditions, the RMT and other altered parameters in MEPs may have been affected by the patients’ general conditions, such as increased intracranial pressure and blood pressure. For example, alterations in motor cortical interneuron excitability related to I1 or late I3 waves under such conditions result in the suppression or enhancement of the MEP response to single TMS (Amassian et al., 1990; Di Lazzaro et al., 2003, 2008). However, in contrast to surgical preoperative cases with severe paresis and consciousness disturbances, the present cases only exhibited targeted motor paresis with small volume, which was therefore favorable for motor function-oriented MEP studies (Fujiki et al., 2021). Second, the relationship between electrophysiological parameters related to motor dysfunction after ICH and its morphological correlation with MRI data [such as diffusion tensor tractography, fractional anisotropy, or apparent diffusion coefficient; Kusano et al. (2009) and Venkatasubramanian et al. (2013)] and the correlations between both modalities in long-term neuronal dysfunction remain unclear. In this regard, the direct link between neurobehavioral features and electrophysiological, morphological, and molecular-level assessments to address safety issues, and 500-Hz high-frequency TBS and MEP amplification induction in the human brain, requires further careful and detailed studies. Indeed, QPS over long durations (up to 30 min) with monophasic high-frequency magnetic or electrical motor cortical stimulation in both humans (Hamada et al., 2013; Shirota et al., 2017) and animals (Müller-Dahlhaus and Vlachos, 2013; Fujiki et al., 2021) can induce long-term potentiation/depression and altered synaptic plasticity. A comparable stimulus potential with a shorter duration was possible with TBS. Therefore, use of current direction manipulation in magnetic brain stimulation-based neuroscience research, such as behavioral experiments or mapping of speech areas (Rogić et al., 2014; Honda et al., 2021), or for future therapeutics (Carmel et al., 2010; Müller-Dahlhaus and Vlachos, 2013), may be beneficial for treating the neurobehavioral deficits related to various neurological disorders.

## Conclusion

In conclusion, 500-Hz high-frequency monophasic TBS using TMS strongly amplified MEPs. Significant correlations were observed among MMT neurological grades, RMT, and 500-Hz QTS-MEP amplitudes. Neurophysiological evaluation of RMT and MEP amplitude may comprise useful surrogate quantitative biomarkers in motor functional evaluations after ICH.

## Data Availability Statement

The raw data supporting the conclusions of this article will be made available by the authors, without undue reservation.

## Ethics Statement

The studies involving human participants were reviewed and approved by Ethics Committee of the School of Medicine, Oita University. The patients/participants provided their written informed consent to participate in this study.

## Author Contributions

MF and YK designed the research paradigm. MF and HF analyzed the data and wrote the manuscript. All authors performed the research, contributed to the article, and approved the submitted version.

## Conflict of Interest

The authors declare that the research was conducted in the absence of any commercial or financial relationships that could be construed as a potential conflict of interest.

## Publisher’s Note

All claims expressed in this article are solely those of the authors and do not necessarily represent those of their affiliated organizations, or those of the publisher, the editors and the reviewers. Any product that may be evaluated in this article, or claim that may be made by its manufacturer, is not guaranteed or endorsed by the publisher.

## References

[B1] Amassian V. E. Quirk G. J. Stewart M. (1990). A comparison of corticospinal activation by magnetic coil and electrical stimulation of monkey motor cortex. *Electroencephalogr. Clin. Neurophysiol.* 77 390–401. 10.1016/0168-5597(90)90061-H 1697531

[B2] Brown M. Avers D. (2018). *Daniels and Worthingnam’s Muscle Testing: Techniques of Manual Examination and Performance Testing.* Philadelphia: Elsevier Health Sciences, 9780323569569.

[B3] Carmel J. B. Berrol L. J. Brus-Ramer M. Martin J. H. (2010). Chronic electrical stimulation of the intact corticospinal system after unilateral injury restores skilled locomotor control and promotes spinal axon outgrowth. *J. Neurosci.* 30 10918–10926. 10.1523/JNEUROSCI.1435-10.2010 20702720 PMC2929360

[B4] Deletis V. Fernández-Conejero I. (2016). Intraoperative monitoring and mapping of the functional integrity of the brainstem. *J. Clin. Neurol.* 12 262–273. 10.3988/jcn.2016.12.3.262 27449909 PMC4960209

[B5] Deletis V. Sala F. (2008). Intraoperative neurophysiological monitoring of the spinal cord during spinal cord and spine surgery: a review focus on the corticospinal tracts. *Clin. Neurophysiol.* 119 248–264. 10.1016/j.clinph.2007.09.135 18053764

[B6] Di Lazzaro V. Oliviero A. Profice P. Pennisi M. A. Pilato F. Zito G. (2003). Ketamine increases human motor cortex excitability to transcranial magnetic stimulation. *J. Physiol.* 547 485–496. 10.1113/jphysiol.2002.030486 12562932 PMC2342642

[B7] Di Lazzaro V. Pilato F. Dileone M. Profice P. Oliviero A. Mazzone P. (2008). The physiological basis of the effects of intermittent theta burst stimulation of the human motor cortex. *J. Physiol.* 586 3871–3879. 10.1113/jphysiol.2008.152736 18566003 PMC2538925

[B8] Fujiki M. Kuga K. Ozaki H. Kawasaki Y. Fudaba H. (2021). Blockade of motor cortical long-term potentiation induction by glutamatergic dysfunction causes abnormal neurobehavior in an experimental subarachnoid hemorrhage model. *Front. Neural Circuits* 15:670189. 10.3389/fncir.2021.670189 33897380 PMC8063030

[B9] Hamada M. Murase N. Hasan A. Balaratnam M. Rothwell J. C. (2013). The role of interneuron networks in driving human motor cortical plasticity. *Cereb. Cortex.* 23 1593–1605. 10.1093/cercor/bhs147 22661405

[B10] Hannula H. Ylioja S. Pertovaara A. Korvenoja A. Ruohonen J. Ilmoniemi R. J. (2005). Somatotopic blocking of sensation with navigated transcranial magnetic stimulation of the primary somatosensory cortex. *Hum. Brain Mapp.* 26 100–109. 10.1002/hbm.20142 15864816 PMC6871677

[B11] Hess G. Donoghue J. P. (1996). Long-term potentiation and long-term depression of horizontal connections in rat motor cortex. *Acta Neurobiol. Exp.* 56 397–405. 10.55782/ane-1996-1143 8787200

[B12] Honda Y. Nakamura S. Ogawa K. Yoshino R. Tobler P. N. Nishimura Y. (2021). Changes in beta and high-gamma power in resting-state electrocorticogram induced by repetitive transcranial magnetic stimulation of primary motor cortex in unanesthetized macaque monkeys. *Neurosci. Res.* 171 41–48. 10.1016/j.neures.2021.02.002 33705847

[B13] Hoogendam J. M. Ramakers G. M. Di Lazzaro V. (2010). Physiology of repetitive transcranial magnetic stimulation of the human brain. *Brain Stimul.* 3 95–118. 10.1016/j.brs.2009.10.005 20633438

[B14] Huang Y. Z. Chen R. S. Rothwell J. C. Wen H. Y. (2007). The after-effect of human theta burst stimulation is NMDA receptor dependent. *Clin. Neurophysiol.* 118 1028–1032. 10.1016/j.clinph.2007.01.021 17368094

[B15] Huang Y. Z. Edwards M. J. Rounis E. Bhatia K. P. Rothwell J. C. (2005). Theta burst stimulation of the human motor cortex. *Neuron.* 45 201–206. 10.1016/j.neuron.2004.12.033 15664172

[B16] Jung N. H. Gleich B. Gattinger N. Hoess C. Haug C. Siebner H. R. (2016). Quadri-pulse theta burst stimulation using ultra-high frequency bursts - A new protocol to induce changes in cortico-spinal excitability in human motor cortex. *PLoS One* 11:e0168410. 10.1371/journal.pone.0168410 27977758 PMC5158069

[B17] Kobayashi S. Matsuyama Y. Shinomiya K. Kawabata S. Ando M. Kanchiku T. (2014). A new alarm point of transcranial electrical stimulation motor evoked potentials for intraoperative spinal cord monitoring: a prospective multicenter study from the Spinal Cord Monitoring Working Group of the Japanese Society for Spine Surgery and Related Research. *J. Neurosurg. Spine.* 20 102–107. 10.3171/2013.10.SPINE12944 24236669

[B18] Kusano Y. Seguchi T. Horiuchi T. Kakizawa Y. Kobayashi T. Tanaka Y. (2009). Prediction of functional outcome in acute cerebral hemorrhage using diffusion tensor imaging at 3T: a prospective study. *AJNR Am. J. Neuroradiol.* 30 1561–1565. 10.3174/ajnr.A1639 19556354 PMC7051627

[B19] Müller-Dahlhaus F. Vlachos A. (2013). Unraveling the cellular and molecular mechanisms of repetitive magnetic stimulation. *Front. Mol. Neurosci.* 6:50. 10.3389/fnmol.2013.00050 24381540 PMC3865432

[B20] Nakamura K. Groiss S. J. Hamada M. Enomoto H. Kadowaki S. Abe M. (2016). Variability in response to quadripulse stimulation of the motor cortex. *Brain Stimul.* 9 859–866. 10.1016/j.brs.2016.01.008 27692928

[B21] Rogić M. Deletis V. Fernández-Conejero I. (2014). Inducing transient language disruptions by mapping of Broca’s area with modified patterned repetitive transcranial magnetic stimulation protocol. *J. Neurosurg.* 120 1033–1041. 10.3171/2013.11.JNS13952 24405070

[B22] Rossi S. Hallett M. Rossini P. M. Pascual-Leone A. (2009). Safety of TMS Consensus Group. *Clin. Neurophysiol.* 120 2008–2039. 10.1016/j.clinph.2009.08.016 19833552 PMC3260536

[B23] Rothwell J. C. (1997). Techniques and mechanisms of action of transcranial stimulation of the human motor cortex. *J. Neurosci. Methods* 74 113–122. 10.1016/s0165-0270(97)02242-5 9219881

[B24] Sekino M. Ohsaki H. Takiyama Y. Yamamoto K. Matsuzaki T. Yasumuro Y. (2015). Eccentric figure-eight coils for transcranial magnetic stimulation. *Bioelectromagnetics* 36 55–65. 10.1002/bem.21886 25399864

[B25] Shirota Y. Dhaka S. Paulus W. Sommer M. (2017). Current direction-dependent modulation of human hand motor function by intermittent theta burst stimulation (iTBS). *Neurosci. Lett.* 650 109–113. 10.1016/j.neulet.2017.04.032 28435045

[B26] Sykes M. Matheson N. A. Brownjohn P. W. Tang A. D. Rodger J. Shemmell J. B. (2016). Differences in motor evoked potentials induced in rats by transcranial magnetic stimulation under two separate anesthetics: implications for plasticity studies. *Front. Neural Circuits* 10:80. 10.3389/fncir.2016.00080 27766073 PMC5052269

[B27] Szelényi A. Kothbauer K. F. Deletis V. (2007). Transcranial electric stimulation for intraoperative motor evoked potential monitoring: stimulation parameters and electrode montages. *Clin. Neurophysiol.* 118 1586–1595. 10.1016/j.clinph.2007.04.008 17507288

[B28] Tamkus A. A. Rice K. S. Kim H. L. (2014). Differential rates of false-positive findings in transcranial electric motor evoked potential monitoring when using inhalational anesthesia versus total intravenous anesthesia during spine surgeries. *Spine J.* 14 1440–1446. 10.1016/j.spinee.2013.08.037 24209393

[B29] Tsutsui S. Iwasaki H. Yamada H. Hashizume H. Minamide A. Nakagawa Y. (2015). Augmentation of motor evoked potentials using multi-train transcranial electrical stimulation in intraoperative neurophysiologic monitoring during spinal surgery. *J. Clin. Monit. Comput.* 29 35–39. 10.1007/s10877-014-9565-7 24532184

[B30] Ushio S. Kawabata S. Sumiya S. Kato T. Yoshii T. Yamada T. (2018). A multi-train electrical stimulation protocol facilitates transcranial electrical motor evoked potentials and increases induction rate and reproducibility even in patients with preoperative neurological deficits. *J. Clin. Monit. Comput.* 32 549–558. 10.1007/s10877-017-0045-8 28710663

[B31] Venkatasubramanian C. Kleinman J. T. Fischbein N. J. Olivot J. M. Gean A. D. Eyngorn I. (2013). Natural history and prognostic value of corticospinal tract Wallerian degeneration in intracerebral hemorrhage. *J. Am. Heart Assoc.* 2:e000090. 10.1161/JAHA.113.000090 23913508 PMC3828779

[B32] Virani S. S. Alonso A. Aparicio H. J. Benjamin E. J. Bittencourt M. S. Callaway C. W. (2021). Hearth Disease and Stroke Statistics 2021-Update: a Report From the American Heart Association. *Circulation* 143 e254–e743. 10.1161/CIR.0000000000000950 33501848 PMC13036842

[B33] Ziemann U. Reis J. Schwenkreis P. Rosanova M. Strafella A. Badawy R. (2015). TMS and drugs revisited 2014. *Clin. Neurophysiol.* 126 1847–1868. 10.1016/j.clinph.2014.08.028 25534482

